# Open questions about the function and evolution of bacterial Min systems

**DOI:** 10.3389/fmicb.2013.00378

**Published:** 2013-12-09

**Authors:** Imrich Barák

**Affiliations:** Department of Microbial Genetics, Institute of Molecular Biology, Slovak Academy of ScienceBratislava, Slovakia

**Keywords:** Min system, bacterial cell division, *Bacillus subtilis* sporulation, *Escherichia coli*, evolution of cell division

Probably one of the most controversial questions about the cell division of rod-shaped bacteria concerns the mechanism that ensures the correct placement of the division septum—mid-cell during vegetative growth but closer to one end during sporulation. In general, bacteria multiply by binary fission in which the division septum forms almost exactly at the cell center. How the division machinery achieves such accuracy is a question of continuing interest. Cell division in *Escherichia coli* and *Bacillus subtilis* are the most thoroughly studied cell division mechanisms. The earliest visible event in cell division is the formation of a Z ring by FtsZ, a tubulin like protein, at the future septum site. The Z-ring appears to be an accurate marker for the position of the division site and is furthermore recognized by set of cell division proteins—the divisome. At least two distinct mechanisms contribute to the placement of the division machinery: nucleoid occlusion and the Min system. The mechanism of Min system action is fundamentally different in both model organisms [reviewed in Barak and Wilkinson (2007)].

## *Escherichia coli* Min system during vegetative growth

Clearly, the best understood Min system is the three protein MinCDE system from *E. coli.* This system was discovered when a set of so-called minicell mutants were observed after the function of any of these three proteins was disrupted (Adler et al., 1967). The mutant cells had difficulty in selecting proper division sites; in some of them the septum formed near the cell poles, pinching off chromosome-free minicells.

**Figure 1:**
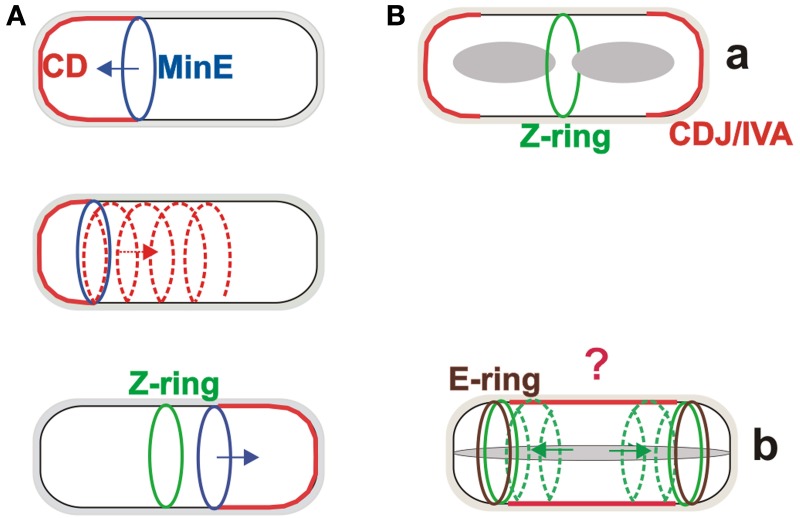
**The Min systems in division site selection in *Escherichia coli* and *Bacillus subtilis***. **(A)** The Min system in *E. coli* oscillates from one pole to the other on a helical trajectory, creating a concentration gradient of the MinC division inhibitor, with the lowest concentration at the mid-cell site where the Z-ring forms. **(B)** The MinCDJ/DivIVA complex (CDJ/IVA) during vegetative growth of *B. subtilis* is localized at the site of septation and cell poles **(a)**. During sporulation, FtsZ relocalizes from the central position toward the poles on helical trajectories where (Ben Yehuda and Losick, 2002), with the help of SpoIIE, it forms the Z-rings. SpoIIE also forms so-called E-rings at both these polar sites, but only one will be used for sporulation septum formation. The role of the Min system during sporulation is not clear (indicated by question mark) but it can likely help to block, at least partially, the mid-cell division site **(b)**.

MinC is a cell division inhibitor which directly binds to FtsZ and is activated by MinD, a membrane-associated ATPase (de Boer et al., 1991). MinE is a topological factor which, together with MinD, provides the localization signals that restrict MinC to zones near the cell poles and away from the cell centre (de Boer et al., 1992). Interestingly, although MinC is localized near the poles, it migrates between the poles in cycles of about 1 min (Raskin and de Boer, 1999). This Min system oscillation system has been observed to follow extended helical arrangements (Shih et al., 2003). It appears to operate in the following way: MinD binds to the membrane as a dimer through its amphipathic helix, and in its ATP bound state. MinE, which also possess an amphiphatic helix, also binds to the membrane where it stimulates the MinD ATPase activity. The resulting MinD–ADP complex monomerizes and dissociates from the membrane. It then resets to its ATP-bound dimer form and re-binds to the membrane some distance away from the membrane bound MinE [reviewed in Rowlet and Margolin (2013)]. The consequence of this extraordinary protein oscillation is that the concentration of MinC is highest at the cell poles and lowest at the mid-cell where the Z-ring appears and, subsequently, the septum forms (Figure 1A).

## *Bacillus subtilis* Min system during vegetative growth

The concentration gradient of the MinC inhibitor in vegetatively growing *B. subtilis* is formed in a strikingly different way. While this model organism has MinC and MinD homologs, it lacks MinE. The localization cue for the MinCD complex is provided by the MinD protein partner MinJ, which is recruited by DivIVA to the site of septation and to the poles (Bramkamp et al., 2008; Patrick and Kearns, 2008; Lenarcic et al., 2009). This creates a more static gradient of MinC, with the largest concentration at the cell poles where septation is prevented (Figure 1Ba). Perhaps surprisingly, *B. subtilis* MinD also has ATPase activity even though it does not drive a rapid oscillation of the protein from pole to pole. On the other hand, fast membrane dissociation and re-association of *B. subtilis* MinD has been observed (Barak et al., 2008). The biological role of this phenomenon is not clear, but the dynamics of MinD localization and reversible membrane binding are integral to the function of both Min systems.

The function of MinD from both *E. coli* and *B. subtilis* is closely linked to the phospholipids of the membrane (Hu and Lutkenhaus, 2001; Barak et al., 2008). Although the phospholipid composition of the membranes of both microorganisms is strikingly different, the oscillation of the *E. coli* Min proteins can still be reproduced after transplantation into *B. subtilis* cells (Jamroskovic et al., 2012). The introduction of this oscillating Min system into *B. subtilis* also inhibits the formation of asymmetric septation during sporulation.

## *Bacillus subtilis* Min system during sporulation

It is possible that the two different Min systems evolved because of the different life cycles of *B. subtilis*, which, in addition to vegetative growth, can undergo sporulation, a simple differentiation process. The first clear, morphological stage of sporulation is formation of a thin asymmetric septum. Before this can happen, however, the Min system function which ensures that the Z-ring appears in the centre of the cell must be overridden. While the complete mechanism of this process is still unknown, partial answers have been provided by the observation that in this stage of development, the function of DivIVA is switched from regulating cell division to allowing proper chromosome segregation to occur in the small part of the cell after asymmetric cell division (the so called forespore). This is likely accomplished by DivIVA switching its binding partner from MinJ to the DNA-binding RacA protein (Ben Yehuda et al., 2003). DivIVA binding sites with RacA and MinJ have recently been described (Van Baarle et al., 2013). It is not known what the MinJ, MinC, and MinD proteins are doing during this stage of sporulation or what their roles are, if any. Although depleting any of these proteins has no detectable effect on sporulation frequency (Cha and Stewart, 1997) it is still not possible to exclude the possibility that the Min system has at least a partial role in sporulation because a sporulation-like septum appears in some *minD* mutant cells to be misplaced from its normal polar site; it forms in either the centre of the cell or nearby (Barak et al., 1998). This suggests that MinCD is likely important for blocking the central septation site during sporulation (Figure 1Bb). In addition, quite different experiments showed that MinCD-dependent repression of SpoIIIE assembly in the forespore is crucial for the proper segregation of the chromosome to the forespore after asymmetric septum formation (Sharp and Pogliano, 2002). At the time of these experiments, the MinJ protein had not yet been discovered, and thus it is not known if these two observed MinCD behaviors are MinJ dependent.

## Evolution of Min systems in bacteria

Min systems exist in most but not all bacterial species, and also in the plastids of higher plants. In some bacteria which lack this system, e.g., *Mycobacterium tuberculosis*, its absence can cause imprecise placement of the Z-ring during cell division. In other bacteria, such as *Caulobacter crescentus*, a completely different mechanism has evolved in which the MipZ protein controls Z-ring formation by creating a bipolar gradient with its minimal concentration at the potential septation site, where FtsZ can assemble. In addition to these mechanisms, in which FtsZ assembly is negatively controlled, there are bacteria in which Z-ring centering is positively regulated. In *Myxococcus xanthus*, the Z-ring is centered by PomZ, which arrives at the mid-cell prior to and independently of FtsZ (Treuner-Lange et al., 2012). Another positively regulated system uses SpoIIE, which stabilizes Z-ring formation at an asymmetric position during sporulation of *B. subtilis*. SpoIIE mutant strains cannot form sporulation septa but nevertheless in a few cells an asymmetrically positioned thick vegetative-like septum can be observed (Illing and Errington, 1991; Barak and Youngman, 1996). Taken together, it seems that nature evolved many different systems for division site recognition and it is likely that new systems are waiting to be discovered.

The existence of two different Min systems is intriguing. The first such system is represented by MinCDE, present in almost all rod-shaped Gram negative bacteria. The second system is formed by the MinCDJ/DivIVA proteins and is present in rod-shaped, mostly endospore forming Gram positive bacterial species. The evolutionary implications of these observations are that bacteria which form endospores will have DivIVA/MinJ rather than MinE as the auxilliary component(s) of MinCD (Jamroskovic et al., 2012). Interestingly, some of the sporulating *Clostrideae* sp. along with *Acetonoma longum* also possess MinE homologs. From the available data it is hard to infer which Min system evolved from which and it has been speculated that both Min systems evolved together in Gram positive bacteria for alternate life cycles of vegetative growth and sporulation. The recent, fascinating description of the cell membrane structures of *A. longum* during sporulation and spore outgrowth showed that during sporulation the inner membrane of the mother cell is inverted and transformed to become the outer membrane of the germinating cell (Tocheva et al., 2011). These findings suggest that sporulation is a mechanism by which the bacterial outer membrane could have arisen. Perhaps *A. longum* is the missing link between Gram positive and Gram negative bacteria, and therefore it is not surprising for it to possess both Min systems. Further work is needed to address whether these Min systems are functional and what interplay, if any, there is between them.
